# Velutin, an Aglycone Extracted from Korean Mistletoe, with Improved Inhibitory Activity against Melanin Biosynthesis

**DOI:** 10.3390/molecules24142549

**Published:** 2019-07-12

**Authors:** Se-Hui Jung, Jaehyun Kim, Juneyong Eum, Jung Won Choe, Hak Hyun Kim, Yun Kee, Kooyeon Lee

**Affiliations:** 1 Department of Bio-Health Technology, College of Biomedical Science, Kangwon National University, Chuncheon 24341, Korea; 2 Division of Biomedical Convergence, College of Biomedical Science, Kangwon National University, Chuncheon 24341, Korea

**Keywords:** microwave-assisted hydrolysis, flavonoid aglycone extract, mistletoe, tyrosinase activity, velutin, zebrafish

## Abstract

The abnormal regulation of melanin synthesis leads to a wide range of pigmentary disorders. Although various melanin biosynthesis inhibitors have been developed, their efficacy and long-term safety needs to be further improved, and thus the goal of this study is to develop promising natural compound inhibitors of melanin biosynthesis. Here, we obtained aglycone flavonoid extract through the microwave-assisted hydrolysis of glycoside extract from Korean mistletoe in acidic condition. The aglycone extract inhibited tyrosinase activity more efficiently with better antioxidant activity than glycoside extract in vitro. The microwave-assisted aglycone extract of mistletoe was further analyzed for in vivo activity, and the results showed the aglycone extract inhibited both early melanocyte development and melanin synthesis more efficiently in zebrafish embryo in a dose-dependent manner. Our in vivo toxicity assay quantitatively measured cell death in zebrafish embryos and showed that the microwave-assisted aglycone extract of mistletoe had no significant effect on cell death (*p* < 0.001), indicating that aglycone extract is more biocompatible than glycoside extract. Furthermore, our in vitro and in vivo analyses successfully identified and characterized velutin, an aglycone of a homoflavoyadorinin B glycoside, as a major inhibitory component in the microwave-assisted mistletoe extract. Ultimately, this study showed that the novel natural compound inhibitor velutin, which was generated through microwave-assisted extraction from mistletoe, improved the efficacy of melanin biosynthesis inhibition with little toxicity.

## 1. Introduction

Melanin is a natural pigment that is synthesized in melanocytes through a multistep process, and it is involved in the determination of skin and hair color [1,2]. Melanin defends cells against harmful light such as UV; however, the abnormal regulation of melanin production is closely related to pigmentary disorders including vitiligo, pityriasis alba, and hyperpigmentation [3]. Melanin synthesis is regulated by multistep catalytic reactions of enzymes such as tyrosinase, tyrosine hydroxylase isoform I, phenylalanine hydroxylase, tyrosinase-related protein 1, and tyrosinase-related protein 2 [4]. Tyrosinase is a copper-containing enzyme that plays a key role in the rate-limiting step of melanin synthesis by the hydroxylation of L-tyrosine to L-3,4-dihydroxyphenylalanine (L-DOPA) and subsequent oxidation of L-DOPA to *o*-quinone (dopaquinone) [5]. Therefore, tyrosinase is a target enzyme for the development of functional cosmetic agents and therapeutics against pigmentary disorders. Certain molecules obtained through chemical synthesis and natural resources, such as hydroquinone, azelaic acid, arbutin, and kojic acid, have been reported to prevent skin pigmentation by inhibiting tyrosinase activity [4,6]. However, due to the cytotoxicity, low efficacy, and low stability of the existing substances, there is still a need for natural substances with improved efficacy and safety.

Flavonoids are a large family of phenolic compound and widely present in the flowers, seeds, leaves, stems, and bark of plants. Flavonoids have emerged as attractive molecules in functional cosmetic ingredients due to their wide range of beneficial biological activities, including their antioxidant, anti-inflammatory, and antimicrobial activities [7,8]. In nature, most flavonoid compounds exist mostly as glycosides, as well as in the form of aglycones [9]. Although it requires further verification, it has been shown that the deglycosylation of flavonoid glycosides to their aglycones through the use of enzymes, heating, and microwave-irradiation improved their biological activities [10,11,12,13]. Microwave-assisted hydrolysis has emerged as a promising technology for the hydrolysis of glycoside extract due to its shorter reaction time and higher efficiency at low cost [14].

Mistletoe (*Viscum album* var. *coloratum*) is a hemiparasitic plant that derives nutritional requirements from oak trees, nettle trees, chestnut trees, as well as others as hosts [15,16]. Mistletoe contains a wide variety of chemical compounds including lectins, viscotoxins, polyphenols, and terpenoids, which have been widely applied as ingredients in herbal medicine throughout Europe and East Asia due to their beneficial biological activities, such as their anticancer, antidiabetic, antihypertensive, and antimelanogenesis activities [13,16,17]. In particular, viscumneoside III and homoflavoyadorinin B, are two flavonoid glycosides that have been extracted from Korean mistletoe and shown inhibitory effects on tyrosinase activity [16]. However, the anti-tyrosinase activity of flavonoid aglycones, which are deglycosylated from flavonoid glycosides of mistletoe, has yet to be examined.

Zebrafish have recently emerged as an attractive animal model for studying melanogenesis [18,19]. This is due to the fact that, in the embryonic development of zebrafish, tyrosinase gene expression and enzyme activity develop within 21 h post-fertilization and melanocytes start to appear from 24 h [20]. Directive 2010/63/EU, an animal protection law established by European Union, allowed for zebrafish larvae to be used for research or toxicity testing up to 96 h post-fertilization (hpf) in embryonic development, which increased the value of the zebrafish model in academic research and commercial development [21,22]. The European Union banned cosmetics-related research involving experimentation on animals through EC Regulation 1223/2009, making zebrafish even more attractive in the cosmetics industry as a resource to replace mammalian animal models [18,19].

In this study, we prepared flavonoid aglycone extract through the microwave-assisted deglycosylation of flavonoid glycosides in crude Korean mistletoe extract under acidic condition and investigated the inhibitory effect of the aglycone extract on melanin biosynthesis in a zebrafish animal model. We identified velutin as a major natural compound inhibitor from the extract and further characterized its effect on melanin biosynthesis inhibition.

## 2. Results and Discussion

### 2.1. Preparation of Flavonoid Aglycon Extract by Microwave-Assisted Hydrolysis of Flavonoid Glycosides

**Figure 1:**
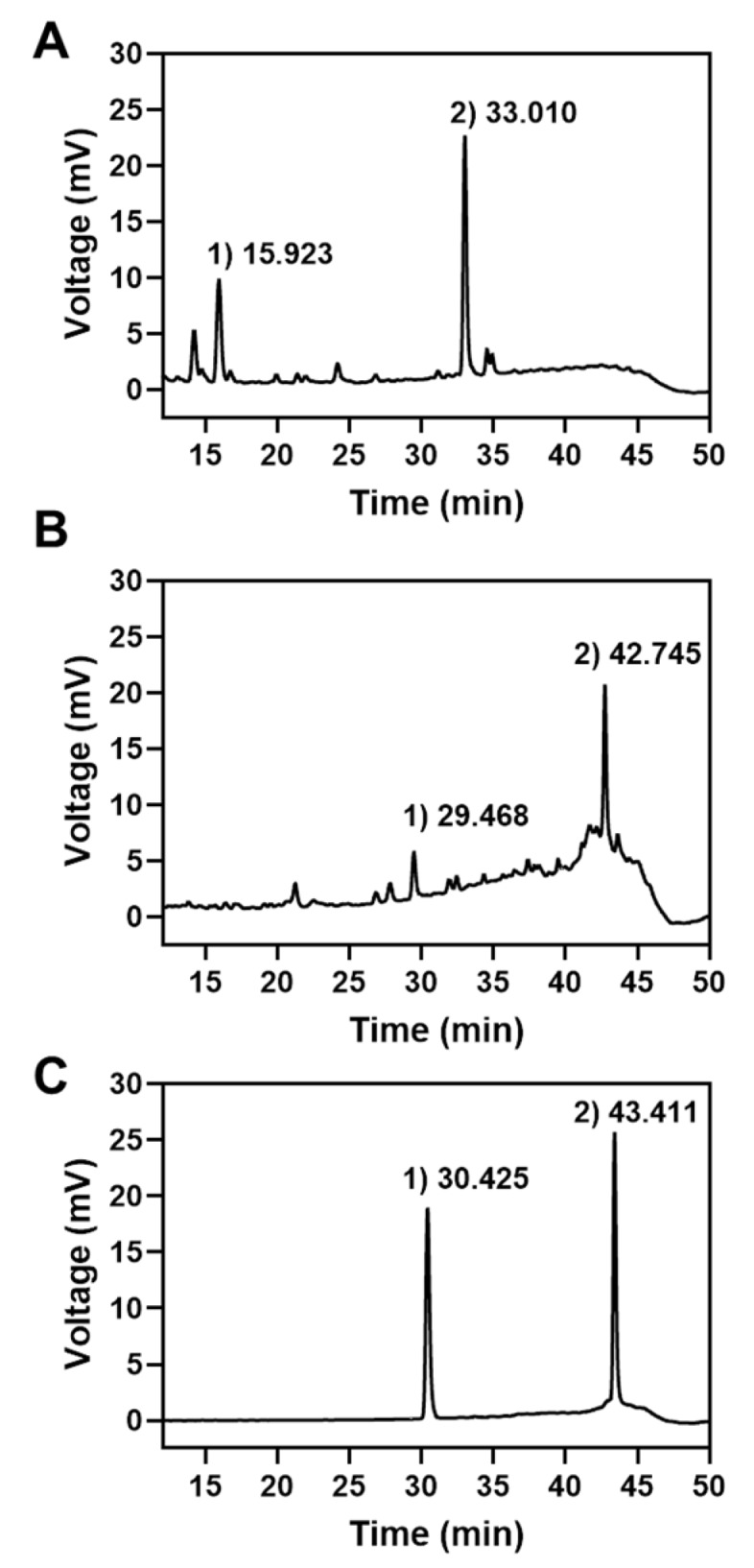
HPLC chromatograms of glycosides-rich (**A**) and aglycones-rich (**B**) extract of the mistletoe and a mixture of homoeriodictyol and velutin (**C**).

In order to assess the potential improvement of natural compound inhibitors in Korean mistletoe, crude ethanol (glycosides-rich) extract of mistletoe was first prepared and further processed through microwave-assisted hydrolysis. The initial HPLC analysis of the crude ethanol extract revealed that viscumneoside III and homoflavoyadorinin B were the two major compounds at the retention times of 15.923 and 33.010 min, respectively (Figure 1A). Next, we optimized the reaction condition of microwave-irradiation for reaction time, temperature, irradiation power, and pH regarding the deglycosylation of flavonoid glycosides in the extract. The crude EtOH extract was irradiated by microwave at 100, 120, or 150 °C at 100 W for 1 h and then analyzed through HPLC under the conditions presented in Table 1. Following irradiation at 120 °C, two major compounds were eluted at an increased retention time (Figure 1B), indicating that the polarities of the two compounds were changed by deglycosylation at 120 °C. The addition of acetic acid to the irradiation reaction increased the deglycolysis rate in a concentration-dependent manner (data not shown). Moreover, the two major compounds produced by microwave-irradiation were eluted at the same retention times as homoeriodictyol and velutin (Figure 1C), which are the deglycosylated forms of viscumneoside III and homoflavoyadorinin B, respectively. Here, we optimized the reaction conditions of the microwave-assisted hydrolysis of mistletoe extract and successfully obtained the flavonoid aglycones-rich extract of mistletoe from glycoside-rich extract (Figure 1B).

### 2.2. Aglycone-Rich Extract from Korean Mistletoe Scavenges ABTS Radical and Inhibits Tyrosinase Activity More Efficiently Than Glycoside-Rich Extract

**Figure 2:**
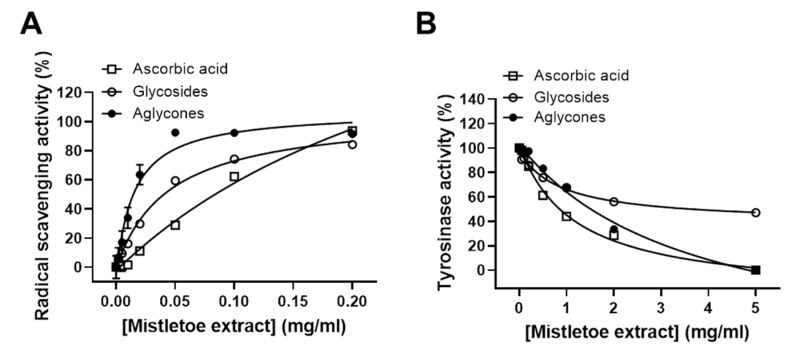
Comparative analysis of glycoside- and aglycone-rich extracts for 2,2′-azino-bis-(3-ethylbenzothiazoline-6-sulphonic acid) (ABTS) radical scavenging and anti-tyrosinase activities. (**A**) Comparative analysis of ABTS radical scavenging activities of glycoside- and aglycone-rich extracts and ascorbic acid. (**B**) Inhibitory effects of glycoside- and aglycone-rich extracts and ascorbic acid against tryosinase activity as determined by measuring the amount of dopachrome produced by catalytic reaction of tyrosinase. Results are expressed as the mean ± SD from three independent experiments.

In order to evaluate the antioxidant activity of the mistletoe extracts, we measured 2,2′-azino-bis-(3-ethylbenthiazoline-6-sulphonic acid) (ABTS) radical scavenging activity within the concentration range from 0.002 to 0.2 mg/mL. Aglycone-rich extract showed concentration-dependent ABTS radical scavenging activity with a maximal scavenging effect of 93% at 0.05 mg/mL, while glycoside-rich extract showed its maximal scavenging effect of 84% at 0.2 mg/mL (Figure 2A).

We also compared the anti-tyrosinase activities of mistletoe glycoside- and aglycone-rich extracts by measuring the amount of dopachrome, which is the product of the tyrosinase-mediated catalytic reaction. Aglycone-rich extract completely inhibited tyrosinase activity at 5 mg/mL, while glycoside-rich extract only inhibited 40% of tyrosinase activity at the same concentration (Figure 2B). These results suggest that the microwave-assisted deglycosylation of flavonoid glycosides to their aglycones in the mistletoe extract may enhance their radical scavenging activity and inhibitory activity against tyrosinase.

### 2.3. Aglycone-Rich Extract Has a More Potent Inhibitory Effect against Melanogenesis in Zebrafish Embryo Than Glycoside-Rich Extract

**Figure 3:**
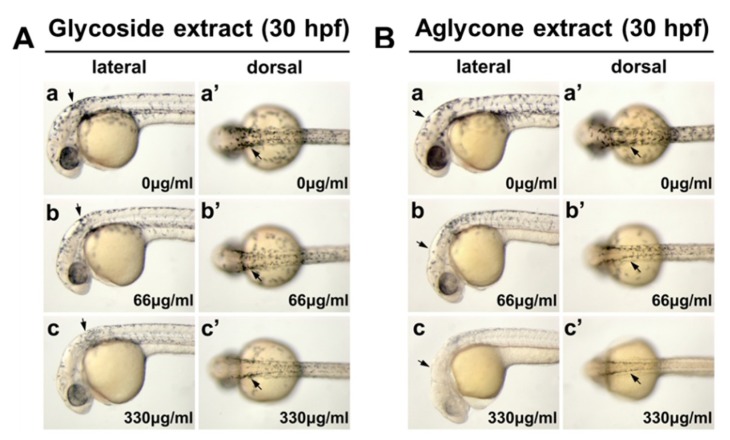
Aglycone-rich extract of mistletoe blocks in vivo melanin synthesis more efficiently than glycoside-rich extract during melanocyte development in zebrafish embryo. Embryos were treated with glycoside extract (**A**) and aglycone extract (**B**) at 0 μg/mL (a,a′), 66 μg/mL (b,b′), and 330 μg/mL (c,c′) in 1% DMSO/E3 media from five hours post-fertilization (hpf), then imaged at 30 hpf. Bright-field images of the embryos are shown in dorsal views (a–c) and lateral views (a′–c′). Melanocytes were developed along most of the anterior-posterior axis (arrow) and near the presumptive retinal pigmented epithelium region (arrowhead). Scale bar 500 μm.

In order to investigate the in vivo recapitulation of our in vitro findings, we first compared the inhibitory effects of the glycoside- and aglycone-rich extracts of mistletoe on melanocyte development in zebrafish embryos at 30 h post-fertilization (hpf). The melanin synthesis in the early melanocyte development in zebrafish embryos was decreased in a dose-dependent manner by both glycoside-rich extract (Figure 3A) and aglycone-rich extract (Figure 3B). The aglycone-rich mistletoe extract reduced melanogenesis more efficiently than the glycoside-rich extract during melanocyte development, and 330 μg/mL aglycone-rich extract showed a particularly dramatic inhibitory effect (Figure 3). This result demonstrated that the microwave-assisted hydrolysis of flavonoid glycosides to their aglycones in the mistletoe extract potentiates the in vivo inhibitory activity on melanogenesis.

### 2.4. Aglycone-Rich Extract of Mistletoe More Efficiently Prevents Melanin Synthesis Than Glycoside-Rich Extract in Zebrafish Embryo

**Figure 4:**
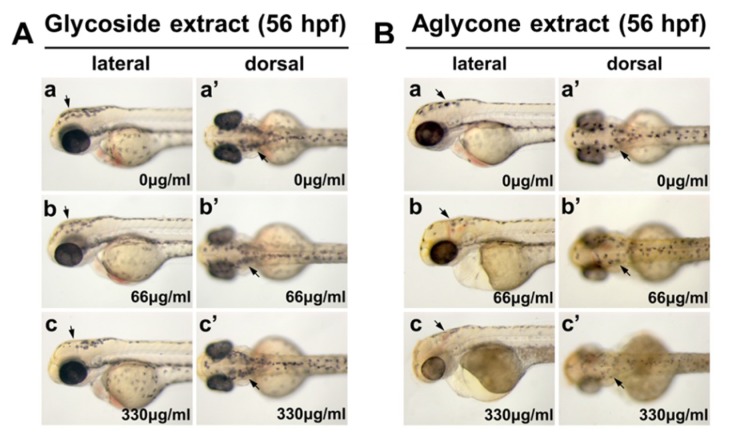
Aglycone extract of mistletoe is a more potent inhibitor on melanin synthesis than glycoside extract in zebrafish embryo. Bright-field images of the embryos are shown in dorsal views (a–c) and lateral views (a′–c′). Embryos were pre-treated with 200 μM phenylthiourea (PTU) in E3 media from 12 h post-fertilization (hpf), washed with 1% DMSO/E3 media at 36 hpf, and then exposed to glycoside-rich extract (**A**) and aglycone-rich extract (**B**) of mistletoe in 1% DMSO/E3 media at 0, 66, and 330 μg/mL until 56 hpf. Melanocytes were distributed along most of the anterior-posterior axis (arrow) and near the presumptive retinal pigmented epithelium region (arrowhead). Scale bar 500 μm.

Next, the capability to maintain the inhibitory activity on in vivo melanin synthesis was examined in this study. Zebrafish embryos were first treated with phenylthiourea (PTU) from 12 hpf, quickly rinsed with 1% dimethyl sulfoxide (DMSO)/E3 medium at 36 hpf, and then treated with mistletoe extracts in 1% DMSO/E3 medium at different concentrations from 36 to 56 hpf. The results indicated that the glycoside-rich extract of mistletoe was slightly effective in inhibiting pigmentation in zebrafish embryos (Figure 4A), but the embryos treated with aglycone-rich extract had a more pronounced effect on preventing melanin synthesis in a dose-dependent manner (Figure 4B). The present data indicate that microwave treatment of the crude ethanol extract of mistletoe increases the in vivo inhibitory effect on melanin synthesis.

### 2.5. Aglycone-Rich Extract of Mistletoe Is More Biocompatible Than Glycoside-Rich Extract

**Figure 5:**
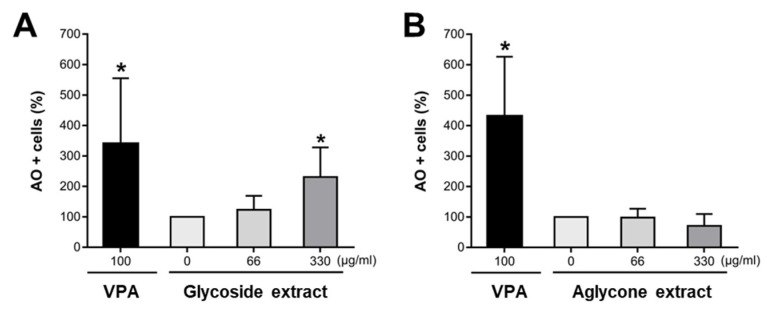
Cytotoxicity tests of glycoside extract and aglycone extract using zebrafish embryo. Embryos were pre-treated with phenylthiourea (PTU) from 12 hpf, switched to media containing glycosides- (**A**) or aglycone-rich extract (**B**) of mistletoe at 36 hpf, and then incubated until 52 hpf. The live 52 hpf embryos were stained with acridine orange (AO) and imaged using a confocal microscope. Cell death was quantitated by calculating the ratio of the number of acridine orange-positive (AO+) cells in the embryos treated with each extract over the number of control embryos treated with vehicle. Results are expressed as the mean ± SD from seven independent experiments, *p < 0.001.

Cytotoxicity is a concern for any potential natural compound, and we thus quantitatively measured in vivo the toxicity of the mistletoe extracts in terms of the anti-pigmentation effect using the acridine orange (AO) staining method that visually labels apoptotic cells as green fluorescence dots in live embryos. As an internal control for different sets of the cell death experiments, we used valproic acid (VPA) as a positive control, which consistently causes abundant cell death in zebrafish embryo. The embryos were exposed to the mistletoe extracts from 36 to 52 hpf, then subjected to live AO staining and imaging analysis using a confocal microscope. Crude ethanol extract containing flavonoid glycosides led to a dose-dependent increase in cell death (Figure 5A), while there was no significant cell death in the embryos treated with the microwave-irradiated extract containing flavonoid aglycones up to 330 μg/mL (Figure 5B). The data indicate that the pigmentation inhibition of the aglycone-rich extract of mistletoe is not attributable to cell death.

### 2.6. Velutin Plays a Major Role in Anti-Pigmentation Effect of Aglycone Extract

**Figure 6:**
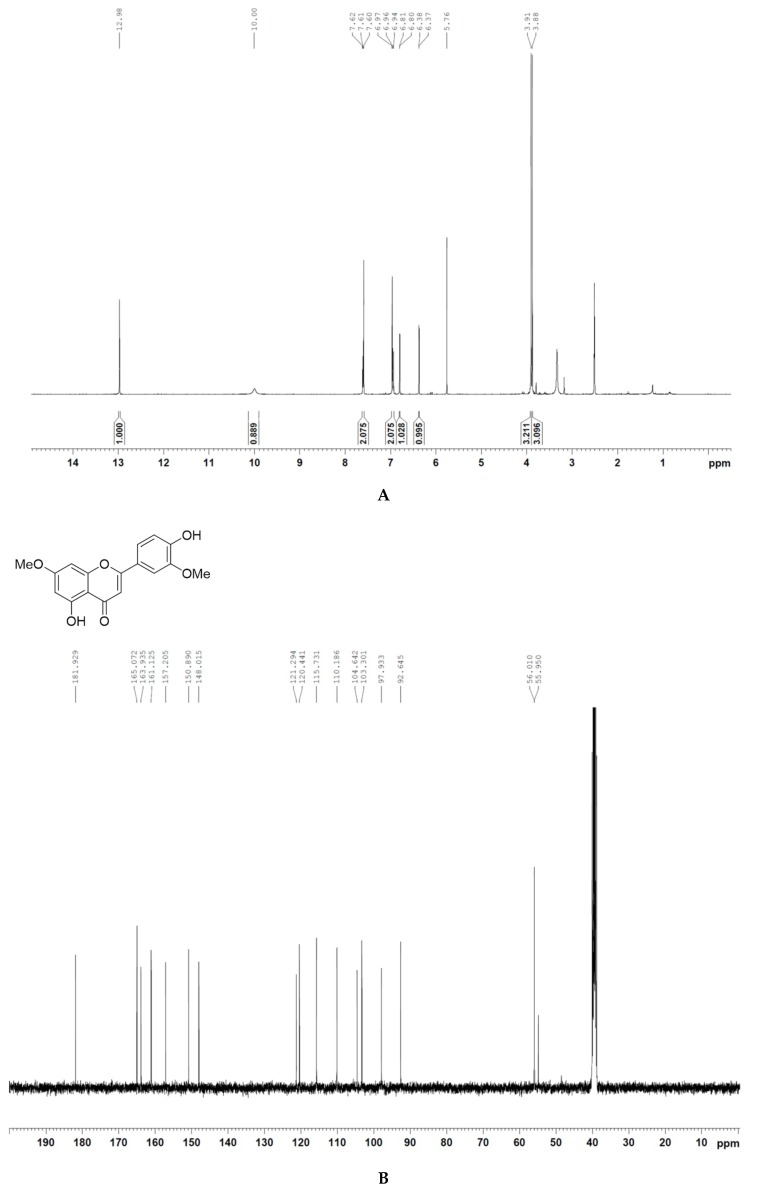
Identification of velutin by ^1^H NMR (**A**) and ^13^C-NMR (**B**) analyses. (**A**) ^1^H-NMR (400 MHz, DMSO): *δ* 12.98 (s, 1H), 10.00 (s, 1H), 7.62–7.60 (m, *J* = 7.24, 2H), 6.97–6.94 (m, *J* = 6.44, 2H), 6.81–6.80 (d, *J* = 2.20, 1H), 6.38–6.37 (d, *J* = 2.24, 1H), 3.91 (s, 3H), 3.88 (s, 3H). (**B**) ^13^C-NMR (100M Hz, DMSO): δ 181.9, 165.0, 163.9, 161.1, 157.2, 150.8, 148.0, 121.2, 120.4, 115.7, 110.1, 104.6, 103.3, 97.9, 92.6, 56.01, 55.9.

**Figure 7:**
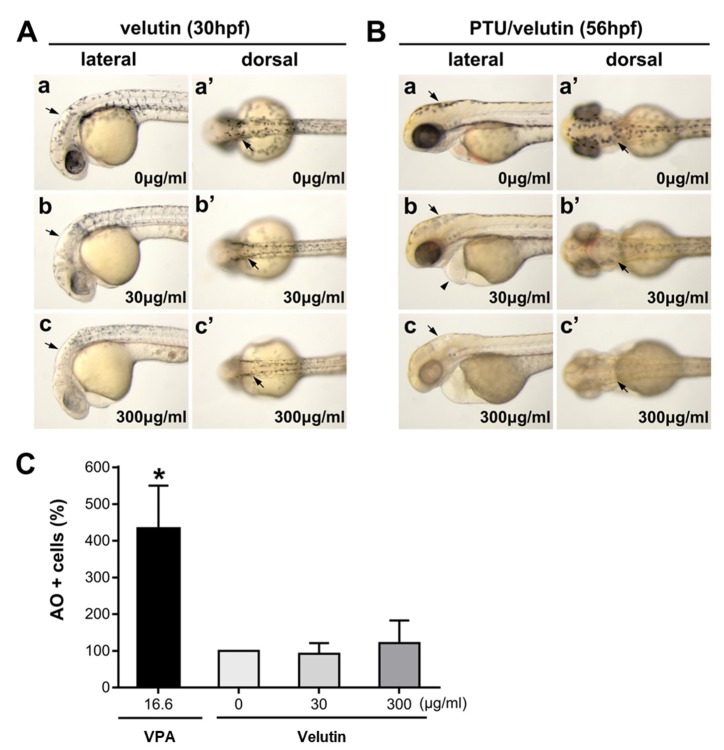
Velutin mimics the anti-pigmentation effect of microwave-assisted aglycone extract of mistletoe in zebrafish embryo. (**A**,**B**) Bright-field images of the embryos are shown in dorsal views (a,b,c) and lateral views (a′,b′,c′). Scale bar 500 μm. (A) Embryos were exposed to 0, 30, and 300 μg/mL of velutin in 1% DMSO/E3 medium from five hours post-fertilization (hpf), then the embryos were imaged at 30 hpf. Velutin reduces pigment cell development in a dose-dependent manner (arrows). (B) Embryos were pre-treated with 200 μM phenylthiourea (PTU) in E3 media from 12 hpf, washed with 1% DMSO/E3 media at 36 hpf, and then exposed to 0, 30, and 300 μg/mL of velutin until 56 hpf. Velutin efficiently inhibits melanin synthesis in a dose-dependent manner (arrows). (**C**) Cell death was quantitated by counting the number of acridine orange-positive (AO+) cells and comparing it to the results for the control with vehicle treatment. There was no significant cell death compared to control embryos. *n* = 5 for each treatment, * *p* < 0.001.

We identified and isolated velutin (Figure 6), an algycone of homoflavoyadorinin B by ^1^H-NMR and ^13^C-NMR analyses, from the microwave extract of mistletoe (Figure 1B,C) and characterized it to investigate its role in the anti-pigmentation effect of the mistletoe extract. Our results showed that velutin efficiently reduced melanogenesis in the melanocyte development of zebrafish embryos (Figure 7A) and maintained an inhibitory effect on melanin synthesis, which was initiated by PTU (Figure 7B) in a dose-dependent manner, mimicking the effect of microwave-assisted flavonoid extract. In addition, according to in vivo cytotoxicity testing through AO staining of zebrafish embryo, velutin did not cause significant cell death at 300 μg/mL (Figure 7C). This indicates that velutin is generated from the microwave-assisted hydrolysis of homoflavoyadorinin B, a glycoside of mistletoe extract, and may serve as a key aglycone flavonoid acting as a natural compound inhibitor on melanin synthesis.

## 3. Experimental Section

### 3.1. Materials

The mistletoe was collected from an oak tree in Jeongseon in South Korea, then dried under ambient condition before being used in this study, as previously described [16]. 2,2′-azino-bis-(3-ethylbenthiazoline-6-sulphonic acid) (ABTS) and 3,4-dihydroxy-L-phenylalanine (L-DOPA) were purchased from Alfa Aesar (Haverhill, MA, USA). Mushroom tyrosinase, 1-Phenyl-2-thiourea (PTU), and Tricaine methanesulfonate (tricaine) were obtained from Sigma-Aldrich (St. Louis, MO, USA).

### 3.2. Microwave-Assisted Hydrolysis of Flavonoid Glycosides

Flavonoid aglycones-rich extract was generated through the microwave-assisted hydrolysis of flavone glycosides-rich extract prepared from the mistletoe, in the manner previously described by our lab [23]. Briefly, dried mistletoe (30 g) was pulverized using a pulverizer (HMF-3250s, Hanil electric, Seoul, Korea), then extracted three times with 70% EtOH (300 mL) at 80 °C for 3 h. The resulting extract was vacuum filtered using Celite 545 (Samchun Pure Chemical Co., Seoul, Korea) and concentrated using a Hei-Vap Advantage rotary evaporator (Heidolph Instruments GmbH & Co. KG, Schwabach, Germany) to obtain 5.2 g glycosides-rich EtOH extract. The microwave-assisted deglycosylation was performed using a microwave reactor, CEM Discover Microwave system (CEM Corporation, Mathews, NC, USA). Briefly, crude EtOH extract (5.2g) was dissolved to a concentration of 200 mg/mL in acetic acid, poured into a reactor, and irradiated by microwave (100 W) for 1 h in a closed system. The temperature of the reactor was kept constant at 120 °C between each heating. The resulting extract was freeze-dried and kept at −80 °C prior to use.

### 3.3. HPLC Analyses

The flavonoid compounds in crude or microwave-irradiated mistletoe extracts were analyzed by HPLC (YL-9100, Young Lin instrument, Anyang, Korea) under the conditions presented in Table 1.

### 3.4. Isolation and Identification of Velutin Using Silica Gel Column Chromatography

The microwave-irradiated crude extract (4.4 g) was subjected to silica gel column chromatography using gradient dichloromethane-methanol (100:1–50:1) to obtain velutin (37 mg). To confirm that the compound is velutin, ^1^H-NMR (400 MHz) and ^13^C-NMR (100 MHz) spectra were recorded on an NMR spectrometer (Bruker Advance 400 MHz spectrometer, Bruker Corporation, Karlsruhe, Germany). Chemical shift values (*δ*) are reported in parts per million relative to the residual signals of this solvent [δ 2.54 for ^1^H (dimethyl sulfoxide-d6), and *δ* 40.5 for ^13^C (dimethyl sulfoxide-d6)].

### 3.5. Determination of ABTS Free Radical Scavenging Activity

In order to evaluate the antioxidant activity of flavonoid glycosides and aglycones extracted from Korean mistletoe, the ABTS radical scavenging activity was determined as described in the previous report with some modifications [24]. First, ABTS^+^ radical cation was obtained by reaction between 7 mM ABTS solution and 2.45 mM K_2_S_2_O_8_ solution for 16 h in the dark. Reaction solution was adjusted with ethanol to yield an absorbance between 0.68 and 0.72 at 734 nm. Then, 100 μL of ABTS solution was added to 100 μL of each extract in the indicated concentration range from 0.002 to 0.2 mg/mL and incubated for 5 min in the dark at room temperature. The absorbance was then measured at 734 nm using a SpectraMax M5 Multi-Mode microplate reader (Molecular Devices, Sunnyvale, CA, USA). The ABTS radical scavenging activity was calculated as follows:

ABTS radical scavenging activity (%) = 1 − (A_sample_/A_control_) × 100. (1)

### 3.6. Tyrosinase Inhibitory Assay

The inhibitory effect of microwave-assisted mistletoe extract on tyrosinase activity was assessed based on quenching of fluorescence by dopachrome produced from the catalytic reaction of tyrosinase [25]. Briefly, each 50 μL of 0.05–5 mg/mL flavonoid extract and ascorbic acid was mixed with 50 μL of 50 U/mL mushroom tyrosinase in 50 mM phosphate buffer (pH 6.8) in a 96-well plate and incubated for 30 min at room temperature. Then, 50 μL of 1 mM L-DOPA was added to each well and further incubated for 10 min at 37 °C. After mixing the solution with 50 μL of 400 ng/mL FITC solution, the fluorescence intensity of the resulting solution was measured using a Synergy H1 Multimode microplate reader from BioTek Instrument, Inc. (Winooski, VT, USA).

### 3.7. Zebrafish Animal Maintenance

Wild type zebrafish (*Danio rerio*), AB strain, was used in this study. The adult zebrafish were maintained in balanced salt water at 27.5 °C with a 14/10 light/dark cycle. The embryos were raised in E3 medium (5 mM NaCl, 0.33 mM MgSO4, 0.33 mM CaCl2, and 0.17 mM KCl) at 28.5 °C.

The animal protocols used in this work were evaluated and approved by the Animal Use and Ethic Committee of the Kangwon National University. They are in accordance with Institutional Animal Care and Use Committee (IACUC) guidelines and the National law for Laboratory Animal Experimentation (Law No. 18.611)

### 3.8. In Vivo Activity Assay of Flavonoid Extract Using Zebrafish Embryos

Each flavonoid extract was dissolved in DMSO and diluted in E3 medium for embryonic treatment to final 1% DMSO. In order to determine the inhibitory activity against melanocyte development, the embryos were treated with glycoside-rich or aglycone-rich flavonoid extract at concentrations of 0, 66, or 330 μg/mL in 1% DMSO/E3 medium from 5 to 30 hpf. Then, bright-field images of the embryos were taken at 30 hpf. In each experiment, five embryos were treated with each extract in 2 mL of E3 medium per well in 24-well plates and incubated at 28.5 °C until undergoing imaging analysis at 30 hpf.

Next, the in vivo inhibitory activity against melanin synthesis was determined as follows: first, 20 embryos were treated with 200 μM PTU in 4 mL of E3 medium in each 30 mm dish at 12 hpf. Next, the embryos were dechorionated at 36 hpf in PTU/E3 medium, washed twice with 1% DMSO/E3 medium, transferred to 24-well plates, and treated with glycoside or aglycone flavonoid extract in 2 mL of 1% DMSO/E3 medium from 36 to 56 hpf in zebrafish embryonic development. Then, imaging analysis was performed at 56 hpf.

### 3.9. Bright Field Imaging of Zebrafish Embryos

Live zebrafish embryos were anesthetized with 0.02% tricaine in E3 medium until they were unresponsive to touch, then they were mounted in 3% methylcellulose for imaging. Bright field images of the embryos were taken using an Olympus SZX16 stereoscope (Olympus, Tokyo, Japan) equipped with an AxioCam GRC camera (Carl Zeiss, Overkochen, Germany).

### 3.10. In Vivo Toxicity Test of Flavonoid Extracts Using Zebrafish Embryos

The toxicity of the flavonoid extracts was evaluated using AO in the manner described previously [26]. Briefly, embryos were pre-treated with 200 μM PTU in E3 medium from 12 hpf, washed with 1% DMSO/E3 medium twice at 36 hpf. The resulting embryos were further grown in glycosides-rich or aglycones-rich flavonoid extract in 1% DMSO/E3 medium until 52 hpf. Next, the embryos were stained with 3 µg/mL AO in E3 medium for 20 min with shaking in the dark and washed twice with E3 medium at 25 °C. The embryos were then anesthetized with 0.02% tricaine/E3 medium and imaged using an LSM700 confocal microscope (Carl Zeiss, Overkochen, Germany). Imaging was performed using 488 nm Ar-laser excitation and a 415–735 nm emission filter. The number of acridine orange positive (AO^+^) cells was counted using the ZEN 2012 program (Carl Zeiss, Oberkochen, Germany). Confocal imaging was conducted at the Korea Basic Science Institute Chuncheon Center.

Cell death was quantitated by calculating the ratio of the number of AO^+^ cells in embryos treated with the flavonoid extracts over the number of control embryos treated with vehicle. The number of AO^+^ cells in the embryos treated with 16.6 μg/mL VPA was used as an internal positive control in each experiment in order to normalize the variation among experiments.

### 3.11. Statistical Analysis

The dopachrome assay results were expressed as the mean ± SD from three independent experiments. AO staining was used to measure in vivo cell death in zebrafish embryos by calculating the ratio of the number of AO^+^ cells in the embryos treated with the flavonoid extracts over the number of control embryos treated with vehicle. The results were expressed as the mean ± SD from five or seven independent experiments, * *p* < 0.001.

Statistical analyses were performed using SPSS 23.0 software (SPSS Inc., Chicago, IL, USA) and Graph-Pad Prism 7.0 (GraphPad Sofware Inc., La Jolla, CA, USA). Five zebrafish embryos per treatment were tested for the effects on melanocyte development and melanin synthesis, and each experiment was repeated for statistical analyses. The difference between the mean values of the control group and the exposed group was analyzed using one-way analysis of variance (ANOVA) followed by a Dunnett’s post hoc test. The threshold for statistical significance for all analyses was *p* < 0.05 (two-tailed).

## 4. Conclusions

In this study, we obtained aglycone flavonoid extract by the microwave-assisted hydrolysis of glycoside extract from Korean mistletoe under acidic condition, and demonstrated that the aglycone extract scavenges ABTS radicals more efficiently and also has a potent inhibitory effect on melanin biosynthesis without significant in vivo toxicity. Furthermore, we newly isolated and characterized velutin, a single component in the mistletoe extract, and showed that velutin strongly mimics the improved inhibitory activity of microwave-assisted flavonoid extract on melanin biosynthesis. Here, we provided a basis for the successful development of a bio beta natural compound inhibitor from a parasite plant to prevent hyperpigmentation.

## Figures and Tables

**Table 1:** Parameters used for HPLC analysis.

Column	TC-C18 (250 × 4.6 mm, Particle Size 5 μm, Agilent, USA)
Flow Rate	0.8 mL/min
Column Temperature	30 ℃
Detection	UV 270 nm
Solvent	0.1% formic acid (A), MeOH (B)
Gradient Elution	0–20 min, 40%–52% B; 20–40 min, 52%–80% B; 5 min, 40% B
